# Antimicrobial susceptibility profiles of thermophilic *Campylobacter* species from human, pig, and chicken feces in Rwanda

**DOI:** 10.3389/fmicb.2025.1570290

**Published:** 2025-08-25

**Authors:** Noel Gahamanyi, Arsene Musana Habimana, Jean Paul Harerimana, Josiane Mukayisenga, Salomon Ntwali, Anathali Umuhoza, Emmanuel Nsengiyumva, Emmanuel Irimaso, Jean Bosco Shimirwa, Cheol-Ho Pan, Erick Vitus Komba, Claude Mambo Muvunyi, Nadine Rujeni, Raghavendra G. Amachawadi

**Affiliations:** ^1^Biology Department, School of Science, College of Science and Technology, University of Rwanda, Kigali, Rwanda; ^2^Rwanda Biomedical Centre, Kigali, Rwanda; ^3^School of Health Sciences, College of Medicine and Health Sciences, University of Rwanda, Kigali, Rwanda; ^4^School of Veterinary Medicine, College of Agriculture, Animal Sciences, and Veterinary Medicine, University of Rwanda, Nyagatare, Rwanda; ^5^Natural Product Informatics Research Center, KIST Gangneung Institute of Natural Products, Gangneung, Republic of Korea; ^6^Tanzania Livestock Research Institute, Dar es Salaam, Tanzania; ^7^Department of Clinical Sciences, College of Veterinary Medicine, Kansas State University, Manhattan, KS, Unites States

**Keywords:** thermophilic *Campylobacter*, antimicrobial resistance, livestock, One Health, Rwanda

## Abstract

Globally, *Campylobacter jejuni* and *C. coli* have been associated with human gastroenteritis. More importantly, there are increasing reports of *Campylobacter* strains that are resistant to commonly used antimicrobials. In Rwanda, the prevalence and the antimicrobial susceptibility profiles of thermophilic *Campylobacter* strains remain underexplored. Since human campylobacteriosis is a foodborne disease with chicken and pigs being among their major reservoirs, this study aimed to determine the prevalence and antimicrobial susceptibility profiles of thermophilic *Campylobacter* species from human, chicken, and pig feces in Rwanda. A total of 385, 337, and 359 human, pig and chicken feces, respectively, were investigated for the presence of *Campylobacter* species. Isolation was done by culture and presumptive colonies were confirmed by the Polymerase Chain Reaction (PCR). The Kirby-Bauer disc diffusion method was employed to determine the susceptibility profiles of obtained isolates against six (06) antimicrobials, namely erythromycin (ERY), ciprofloxacin (CIP), streptomycin (STR), gentamicin (GEN), tetracycline (TET), and chloramphenicol (CHL). The used antimicrobials include drugs of choice or alternative treatment for human campylobacteriosis. The overall prevalence of thermophilic *Campylobacter* was 7.0% (27/385) in humans, 7.1% (24/337) in pigs, and 32.0% (115/359) in chicken. *C. jejuni* was the predominant species in all hosts with detection frequencies of 92.6%, 66.7%, and 73.9% in humans, pigs, and poultry, respectively. Increased resistance rates to ERY (70.1–92.4%) and STR (68.2–88.0%) were observed particularly among chicken isolates. Multi-drug resistance (MDR) was observed among the isolates, with the highest rates observed in chicken isolates (88.0%). Proportions of MDR among pig (40.9%) and human (40.7%) isolates were more or less similar. These findings highlight the presence of thermophilic *Campylobacter* strains in humans and livestock which are resistant to commonly used antimicrobials. Based on the potential of interspecies transmission, it is recommended to adopt a One Health approach to curb antimicrobial resistance. Further genomic analysis will shed more light on the transmission and drug resistance patterns.

## Introduction

*Campylobacter* spp. are major foodborne pathogens responsible for zoonotic gastroenteritis worldwide (Scallan et al., 2011) with *C. jejuni* and *C. coli* accounting for 95% of human campylobacteriosis cases (Badjo et al., 2024; Acheson and Allos, 2001). Different reservoirs have been associated with campylobacteriosis but poultry has been linked to around 70% of human campylobacteriosis (Epps et al., 2013; Boes et al., 2005). Pigs are considered the primary host for *C. coli* which is the second most prevalent species linked to human campylobacteriosis after *C. jejuni* (Wagenaar et al., 2013; Georges-Courbot et al., 1986). Overall, Clinical symptoms of *Campylobacter* infections include abdominal pain, diarrhea, vomiting, chills, and fever (Same and Tamma, 2018). The transmission of *Campylobacter* to humans occurs through the consumption of raw or undercooked meat, especially poultry, contaminated milk and water, and direct contact with farm animals (Khairullah et al., 2024; Akase et al., 2024; Igwaran and Okoh, 2020).

The escalating incidence of campylobacteriosis, particularly in low- and middle-income countries (LMICs), presents a significant public health challenge. Africa is estimated to have the world's highest incidence of campylobacteriosis (French et al., 2024). The latter is hyper-endemic in sub-Saharan Africa (SSA) due to factors such as poor sanitation, limited access to clean water, and close contact with animals in domestic and agricultural settings (Olum et al., 2023). Human campylobacteriosis often presents symptoms similar to other gastrointestinal infections, which explains its frequent exclusion from the diagnostic and reporting processes (Paintsil et al., 2023; Gahamanyi et al., 2020a; Asuming-Bediako et al., 2019). Despite its significant occurrence in the food chain and strong association with diarrheal cases in children (Coker et al., 2002; Gölz et al., 2018), campylobacteriosis has generally received modest attention.

Indiscriminate use of antibiotics in human and veterinary medicine has led to an increased resistance of *Campylobacter* strains to the drugs of choice. Indeed, both macrolides (erythromycin or azythromycin) and fluoroquinolone (ciprofloxacin) were used as drugs of choice (Wieczorek and Osek, 2013; García-Fernández et al., 2018), but increasing resistance of *Campylobacter* to ciprofloxacin has led to the adoption of erythromycin as the best choice (Dai et al., 2020). Alternative treatment include aminoglycosides and tetracyclines (Koolman et al., 2015; Reddy and Zishiri, 2017). Furthermore, the World Health Organization (WHO) has classified fluoroquinolone-resistant *Campylobacter* as a priority pathogen requiring the development of new antibiotics (Gahamanyi et al., 2020b). We previously showed an increased resistance to ciprofloxacin and erythromycin in Ghana and Tanzania among *Campylobacter* of human origin (Gahamanyi et al., 2020a) while Kenya recorded an increased resistance against tetracycline and ciprofloxacin in *Campylobacter* from chicken (Asuming-Bediako et al., 2019). Also, a study reported a high prevalence of multidrug-resistant (MDR) *Campylobacter* isolates from humans and poultry in Tanzania and Kenya (French et al., 2024). Thus, antimicrobial-resistant *Campylobacter* strains are becoming more prevalent in the East African region (Gahamanyi et al., 2020a; Asuming-Bediako et al., 2019; Zachariah et al., 2021).

In Rwanda, *Campylobacter* species were identified in human stool (Kabayiza et al., 2014) and environmental samples (Ssemanda et al., 2018), but the prevalence in livestock is not fully documented. Moreover, studies on antimicrobial susceptibility profiles of *Campylobacter* isolates are scarce, possibly due to inherent difficulties associated with its culture (Mileng et al., 2021) or the fact that it is not on the list of commonly suspected bacteria in clinical settings. Therefore, the objective of the study was to determine the prevalence and antimicrobial susceptibility profiles of thermophilic *Campylobacter* species from humans, chicken, and pigs in Rwanda. The ultimate goal is to generate data that will be used to design a One Health control strategy for campylobacteriosis and associated antimicrobial resistance.

## Materials and methods

### Study area

The study was conducted in three (03) different districts of Rwanda, namely Nyarugenge (Kigali city), Huye, and Gisagara Districts (Southern Province). Clinical samples were collected from Kigali University Teaching Hospital (CHUK), Nyarugenge District Hospital, Muhima and Biryogo Health Centers, while pig and poultry samples were collected from the largest farms located in the Southern Province (Huye and Gisagara Districts).

### Study design

This study adopted a cross-sectional design and was conducted from March 2024 to August 2024. The study used a purposive sampling strategy. The human aspect targeted individuals presenting at the hospital with abdominal pain and/or diarrhea.

### Sample size determination

The sample size was determined using Cochran's Formula as follows:

Formula: *n* $=\frac{Z^{2}p\left(1-p\right)}{e^{2}}$. Where n is the sample size; *Z*^2^ is the *Z* score (*Z* = 1.96); p is the expected prevalence; and *e*^2^ is the desired level of precision or margin of error. Since the confidence interval is 95%, the error margin is 0.05 or 5%. For humans, *p* = 0.5 used when the true prevalence is unknown was chosen and the sample size being *n* = 385. For pigs, the sample size of 337 was calculated based on previous prevalence (*p* = 0.325) (Kashoma et al., 2015). For chicken, the sample size was 376 based on previous prevalence (*p* = 0.425) (Chuma et al., 2016).

### Sample collection and *Campylobacter* isolation

Human stool samples (*n* = 385) were collected from CHUK (*n* = 21), Nyarugenge DH (*n* = 63), Muhima HC (*n* = 58), and Biryogo HC (*n* = 243). Pig feces (*n* = 337) were collected from Tumba (*n* = 56), Ngoma (*n* = 56), Maraba (*n* = 56), Kigoma (*n* = 56), and Ruhashya (*n* = 56) sectors of Huye and Save sector of Gisagara district (*n* = 57). Chicken feces (*n* = 376) were collected from poultry farms located in Ruhashya (*n* = 130) and from one farm in Ngoma (*n* = 246) of Huye district. In all sites, the collected samples were placed into stool collection containers labeled with the site and geographical locations. Approximately five (05) grams of fecal samples were collected and transported in a cooler box to the Microbiology laboratory of the University of Rwanda, College of Science and Technology, for further processing.

Upon reaching the laboratory, human stool samples were inoculated into the Nutrient Broth No.2 (CM0067, Oxoid, Basingstoke, UK) supplemented with Preston *Campylobacter* selective Supplements SR0204 and SR0232E (Oxoid Ltd, Basingstoke, Hampshire, England), and 5% defibrinated Sheep Blood (SR0051B; Oxoid Ltd, Basingstoke, Hampshire, England). Tubes were incubated at 42 °C for 24 h under microaerophilic conditions generated with CampyGen™ sachets CN0025A (Oxoid Ltd, Basingstoke, Hampshire, England) before being subcultured onto modified Charcoal Cefoperazone Deoxycholate Agar (mCCDA; Oxoid Ltd, Basingstoke, Hampshire, England) as previously described (Srijan et al., 2013). Pig and chicken feces were immediately inoculated onto mCCDA supplemented with SR0155E (Oxoid Ltd, Basingstoke, Hampshire, England). Plates were incubated at 37 °C for 48 h (Guévremont et al., 2006). Presumptive colonies of *Campylobacter* (moistened, gray, flat, and tendency to spread) were sub-cultured onto blood agar supplemented with 5% (v/v) of defibrinated sheep blood and incubated at 37 °C for 48 h under microaerophilic conditions as previously described (Guévremont et al., 2006). Obtained pure colonies were further characterized with Gram staining, cellular morphology, catalase, and oxidase tests (Modi et al., 2015). *Campylobacter* isolates were preserved in Mueller Hinton Broth (MHB) supplemented with 25% glycerol and kept at −80 °C until further processing as previously described (Yildiz et al., 2024).

### DNA extraction and Polymerase chain reaction (PCR)

Genomic DNA was extracted from pure colonies using the Quick-DNA TM Fecal/Soil Microbe Miniprep Kit (ZYMO Research, USA) according to the manufacturer's instructions. Extracted DNA was quantified using a quantusTM fluorometer while the quality of the extracted DNA was assessed with a nanodrop (EzDrop 1000 micro-volume spectrophotometer). Extracted DNA samples were of good quality and quantity for further processing. DNA samples were kept at −20 °C before PCR was performed.

After DNA extraction, a multiplex-PCR using genus-specific primers (C412F; C1228R)*, C. jejuni* and *C. coli* primers was carried out as previously described (Yamazaki-Matsune et al., 2007). The choice of primers was made with a focus on their ability to specifically identify the genus and species of *Campylobacter* (Linton et al., 1997; Pajaniappan et al., 2008). The PCR mixture was 25 μl, and it comprised 12.5 μl of 2X Master Mix (New England Biolabs), 1 μl of each primer (10 μM), 1.5 μl of template DNA (20 μg/ml), and 9 μl of sterile deionized water. UltraPure™ DNase/RNase-Free Distilled Water (Invitrogen) was used as negative control for each PCR run. The amplification conditions involved one cycle at 95 °C for 5 min for denaturation, followed by 35 cycles, each consisting of 94 °C for 30 s, 60 °C for 45 s for annealing, and 72 °C for 45 s, with a final extension at 72 °C for 7 min (Gahamanyi et al., 2021). PCR products were kept at 4 °C before analyzed by gel electrophoresis. The bands of the amplification products were compared to the NEB 100 bp DNA ladder (New England Biolabs). Gel electrophoresis was performed for band visualization of PCR products. A 2% (v/v) agarose gel stained with gel red suspended in 1X TAE buffer was utilized. Samples were loaded into wells using 6X blue-orange loading dye, and bands of PCR products were observed with a UV transilluminator at 161 bp, 502 bp, 816 bp for *C. jejuni, C. coli*, and *Campylobacter* genus, respectively. For quality control, *C. jejuni* strain (ATCC^®^ 33560™) and *C. coli* strains (ATCC^®^ 33559™) were used.

### Antimicrobial susceptibility testing

Antimicrobial susceptibility testing (AST) was conducted using the disc diffusion method on Muller Hinton Agar (MHA; Oxoid Ltd., Basingstoke, UK) in accordance with the guidelines set by the Clinical Laboratory Standards Institute (CLSI) (2024) and the European Committee for Antimicrobial Susceptibility Testing (EUCAST) for *Campylobacter* isolates from human samples and livestock^1^, respectively. EUCAST lacks Gentamicin and Chloramphenicol guidelines for *C. jejuni* and *C. coli* and therefore, Enterobacteriales breakpoints were used. For livestock, since Streptomycin was missing from the EUCAST guidelines, CLSI-Enterobacterales guidelines were used. Various antibiotics, including Ciprofloxacin (CIP, 5 μg), Erythromycin (ERY, 15 μg), Tetracycline (TET, 30 μg), Streptomycin (STR, 10 μg), Gentamicin (GEN, 10 μg), and Chloramphenicol (CHL, 30 μg) were used to assess the AST profiles of isolated *Campylobacter*. However, due to limited stock, TET was not used for *Campylobacter* isolates from chicken.

Briefly, preserved *Campylobacter* isolates were revived onto Blood Agar and sub-cultured onto the same media to obtain pure colonies free from glycerol. Obtained pure colonies were suspended in sterile normal saline, and turbidity was adjusted to 0.5 McFarland standard. Using sterile glass spreaders, bacterial suspension was spread over MHA supplemented with 5% defibrinated sheep blood and 20 mg/L β-NAD. Using forceps, antibiotic discs were evenly distributed over the plates ensuring a spacing of at least 24 mm. Plates were then incubated at 42 °C for 24 h in a microaerophilic environment as previously described (Strakova et al., 2024). After incubation, the inhibition zones were measured using a ruler. Resistance to three or more classes of antibiotics was referred to as multidrug resistance (MDR) as previously described (Ocejo et al., 2019).

### Data analysis

Microsoft Excel was used for data entry and cleaning while SPSS version 27 was used for statistical analysis. Descriptive statistics including proportions of antibiotic resistance, and attribute frequencies, were computed. Antimicrobial susceptibility testing was done based on cultural characteristics. Samples harboring two species of *Campylobacter* (*C. jejuni* and *C. coli*) were removed from the final AST analysis. To assess the association between source of sample and *Campylobacter* species, a chi-square test of independence was performed. This test was performed at a 95% confidence level with a significance threshold set at α = 0.05.

## Results

### Prevalence of *Campylobacter* in different compartments

The prevalence of *Campylobacter* varied across different sampling sites and sample types. In humans, the overall positivity rate was 7.0%, with Biryogo HC recording the highest prevalence at 8.6%, followed by Nyarugenge DH (6.3%) and Muhima H.C. (3.4%). No positive cases were detected at CHUK. In pigs, the prevalence was 7.1%, and Save exhibited the highest positivity rate of 21.1%, followed by Kigoma and Ruhashya (8.9%) and Maraba (3.6%). No positive cases were detected in samples from Tumba and Ngoma. Chicken samples had the highest prevalence of *Campylobacter* at 32.0%, with positivity rates of 34.9% in Ngoma and 26.9% in Ruhashya (Table 1).

**Table 1:** Distribution of samples, and isolation frequencies of *Campylobacter* from humans, pigs, and chicken in Rwanda (2024).

**Sample type**	**Sampling site**	**Total samples tested**	**Number of positives**	**Percentage of positive samples**
Human	CHUK	21	0	0.0
	Biryogo HC	243	21	8.6
	Muhima HC	58	2	3.4
	Nyarugenge DH	63	4	6.3
	**Total (Human)**	**385**	**27**	**7.0**
Pig	Save	57	12	21.1
	Kigoma	56	5	8.9
	Maraba	56	2	3.6
	Tumba	56	0	0.0
	Ruhashya	56	5	8.9
	Ngoma	56	0	0.0
	**Total (Pig)**	**337**	**24**	**7.1**
Chicken	Ruhashya	130	35	26.9
	Ngoma	229	80	34.9
	**Total (Chicken)**	**359**	**115**	**32.0**

*Campylobacter* isolates were identified as *C. jejuni* and *C. coli* by the polymerase chain reaction. Findings showed that the predominant species was *C. jejuni* with detection rates varying between 66.7 and 92.6%, while *C. coli* was detected at rates varying between 7.4 and 27.3% (Table 2). The chi-square test of independence revealed a significant association between sample source and *Campylobacter* species (*p*-value of 0.023). We also identified both species (*C. jejuni* and *C. coli*) in two isolates (8.3%) from pigs and 23 (20.0%) from chicken.

**Table 2:** The detection frequencies of *Campylobacter* species in the three hosts.

**Species**	**Human (*n* = 27)**	**Pig (*n* = 22)**	**Chicken (*n* = 92)**	***P*-value**
*C. coli*	2 (7.4%)	6 (27.3%)	7 (7.6%)	0.023
*C. jejuni*	25 (92.6%)	16 (72.7%)	85 (92.4%)	

### Antimicrobial susceptibility profiles of *Campylobacter* species from human, pigs, and chicken

Antimicrobial susceptibility testing (AST) was subsequently performed on isolates from the two species (*C. jejuni* and *C. coli*) identified by PCR. *Campylobacte*r isolates from all hosts showed higher resistance to ERY and STR. In chicken, *C. coli* demonstrated resistance rates of 100, 85.7, and 71.4% to CIP, ERY, and STR, respectively. Among pig isolates, *C. coli* exhibited high resistance to ERY (83.3%) and CIP (66.7%), while lower resistance rates were observed for gentamicin (GEN, 33.3%) and chloramphenicol (CHL, 16.7%). In human isolates, high resistance was noted against STR (100%) and ERY (50%), whereas no resistance was detected for GEN, CIP, CHL, and tetracycline (TET; Table 3).

**Table 3:** Antimicrobial resistance profiles of identified *C. jejuni* and *C. coli* species from the three hosts.

**Antimicrobial/species**	**Human (*****n*** = **27)**			**Pig (*****n*** = **22)**			**Chicken (*****n*** = **92)**		
	***C. coli***, ***n*** = **2(%)**	***C. jejuni***, ***n*** = **25 (%)**	**Total**	***C. coli***, ***n*** = **6 (%)**	***C. jejuni***, ***n*** = **16(%)**	**Total**	***C. coli***, ***n*** = **7(%)**	***C. jejuni***, ***n*** = **85 (%)**	**Total**
GEN	0 (0.0)	6 (24)	6 (22.2)	2 (33.3)	3 (18.9)	5 (22.7)	4 (57.1)	58 (68.2)	62 (67.4)
CIP	0 (0.0)	7 (28)	7 (25.9)	4 (66.7)	2 (12.5)	6 (27.3)	7 (100)	80 (94.1)	87 (94.6)
ERY	1 (50.0)	19 (76)	20 (70.1)	5 (83.3)	11 (68.8)	16 (72.7)	6 (85.7)	79 (92.9)	85 (92.4)
STR	2 (100)	21 (84)	23 (85.2)	3 (50.0)	12 (75.0)	15 (68.2)	5 (71.4)	76 (89.4)	81 (88.0)
CHL	0 (0.0)	2 (8.0)	2 (7.4)	1 (16.7)	0 (0.0)	1 (4.5)	2 (28.6)	25 (29.4)	27 (29.3)
TET	0 (0.0)	8 (32.0)	8 (29.6)	3 (50.0)	3 (18.9)	6 (27.3)	–	–	–

The susceptibility testing of Campylobacter isolates from chickens against TET was not conducted due to a limitation in the availability of antimicrobial disks.

For *C. jejuni*, high levels of resistance were observed in chicken isolates, with rates of 94.1, 92.9, and 89.4% for CIP, ERY, and STR, respectively. In pig isolates, notable resistance was recorded for STR (75%) and ERY (68.8%). Among human isolates, resistance was high for ERY (76%) and STR (84%; Table 3).

Of the 92 *Campylobacter* isolates from chicken, 91 (98.9%) were resistant to at least one antimicrobial agent. Similarly, 25 (92.6%) out of 27 *Campylobacter* isolates from humans and 20 (90.9%) out of 22 *Campylobacter* isolates from pigs exhibited resistance to at least one antimicrobial agent (Table 4).

**Table 4:** Antimicrobial resistance profiles of *Campylobacter* from the three hosts.

**Resistance pattern**	**Chicken isolates (*n* = 92)**	**Pig isolates (*n* = 22)**	**Human isolates (*n* = 27)**
No resistance	1 (1.1%)	2 (9.1%)	2 (7.4%)
Resistance to one drug	5 (5.4%)	4 (18.2%)	5 (18.5%)
Resistance to two drugs	5 (5.4%)	7 (31.8%)	9 (33.3%)
GEN-CIP-ERY	60 (65.2%)	2 (9.1%)	5 (18.5%)
GEN-CIP-ERY-STR	58 (63.0%)	1 (4.5%)	5 (18.5 %)
GEN-CIP-ERY-STR-CHL	23 (25.0%)	0 (0.0%)	0 (0%)
CIP-ERY-STR-CHL	26 (28.3%)	0 (0.0%)	0 (0%)
CIP-ERY-STR	76 (82.6%)	2 (9.1%)	0 (%)
ERY-STR-CHL	27 (29.3%)	0 (0.0%)	1 (3.7%)
GEN-ERY-STR	59 (64.1%)	2 (9.1%)	5 (18.5%)
GEN-ERY-STR-CHL	23 (25.0%)	0 (0.0%)	0 (0%)
GEN-STR-CHL	23 (25.0%)	0 (0.0%)	0 (0%)
CIP-STR-CHL	12 (13.0%)	0 (0.0%)	0 (0%)
GEN-ERY-TET	–	2 (9.1%)	4 (14.8%)
Overall MDR^*^	**81 (88.0%)**	**9 (40.9%)**	**11 (40.7%)**

* An isolate was considered as multidrug resistant (MDR) if it exhibited resistance to three or more classes of antimicrobial agents. GEN, gentamicin; CIP, ciprofloxacin; ERY, erythromycin; STR, streptomycin; CHL, chloramphenicol; TET, Tetracycline.

The MDR profiles of *Campylobacter* isolates against tested antimicrobials were also evaluated. Overall, MDR was observed in 88.0% (*n* = 81), 40.9% (*n* = 9), and 40.7% (*n* = 11) isolates from chicken, pigs, and humans, respectively. In chicken, the highest MDR rate (82.6%) was observed against CIP-ERY-STR. In pigs, the highest MDR rate of 9.1% was observed against GEN-CIP-ERY, CIP-ERY-STR, GEN ERY-STR, and GEN-ERY-TET. For human isolates, the highest MDR rate (18.5%) was observed against GEN-CIP-ERY-STR, GEN-CIP-ERY, and GEN-ERY-STR (Table 4).

## Discussion

The current study observed varying frequencies of isolation of thermophilic *Campylobacter* from human, pig, and chicken samples. The highest prevalence of *Campylobacter* was observed in chicken (32%), while the prevalence of the bacterium in humans (7%) and pigs (7.1%) was similar. The findings of this study suggest that the poultry-human transmission route could be more important than the pig-human transmission. Furthermore, this is in line with findings from our systematic review indicating that chicken had the highest prevalence of *Campylobacter* among known reservoirs in Africa (Gahamanyi et al., 2020a). Indeed, chicken are known to be the primary reservoir for *Campylobacter* (de Zoete et al., 2007) due to their high body temperature (42 °C) favorable for the bacteria's growth (Sibanda et al., 2018). A previous human study in Rwanda reported a higher *Campylobacter* prevalence of 15.5% (Kabayiza et al., 2014). The discrepancy may be a result of the diagnostic methods used (PCR vs. culture) and the age of patients (infants and younger children vs. adults). The PCR is more sensitive than culture and children are more at risk of *Campylobacter* of infections especially in LMICs. Nevertheless, our findings are comparable to findings reported in Ethiopia (Worku et al., 2024) but lower than the one reported in Tanzania (Komba et al., 2015). Contrarily, the prevalence recorded in this study was higher than the one reported in Burkina Faso (Sangaré et al., 2012). Since humans acquire *Campylobacter* through food/drink contamination from reservoir animals (poultry, pigs, cattle), the observed country-level differences may be due to the varying food chains, feeding habits, and differing sanitation levels. For pigs, the prevalence of *Campylobacter* obtained in this study was slightly higher than 2.3% reported in South Africa (Jonker and Picard, 2010), but significantly lower than 32.5% reported in Tanzania (Kashoma et al., 2015). These disparities likely reflect differences in production systems, dietary practices, and standards of hygiene. For instance, the Rwandan pig food chain is less developed compared to the one in Tanzania.

*Campylobacter jejuni* was the predominant species in all three hosts with 92.6, 72.7, and 66.7% in humans, chicken, and pigs, respectively. The prevalence of *C. coli* was higher in pigs (27.3%), while co-occurrence of both species was recorded in 20 and 8.3% of chicken, and pig, respectively. Generally, human campylobacteriosis is associated with *C. jejuni* at 80–85%, while the percentage of infection due to *C. coli* is between 10 and 15% (Bullman et al., 2012). Worldwide, *C. jejuni* is more common in poultry, while *C. coli* is predominant in pigs (Harvey et al., 1999). The increased prevalence of *C. jejuni* can be linked to the fact that it survives better at low temperatures than *C. coli* (Ortiz et al., 2024). It is important to note that *C. jejuni* is equipped with virulence genes and is associated with higher pathogenesis compared to *C. coli* (Thakur et al., 2010). It is also not surprising to obtain both *C. jejuni* and *C. coli* as previously described (Liao et al., 2022).

The current study assessed the antimicrobial susceptibility profiles of *Campylobacter* isolates. In general, over 90% of *Campylobacter* isolates from all three (03) hosts exhibited resistance to at least one antimicrobial agent. Higher resistance rates ranging from 70.8 to 85.2% against STR and 74.1 to 83.5% against ERY were observed in this study. The resistance of *Campylobacter* isolates from chicken to CIP was 93.9%. Both CIP and ERY are drugs of choice for the treatment of human campylobacteriosis (Harvey et al., 1999), but raising resistance levels call for alternative treatment strategies (Dai et al., 2020). Erythromycin and azithromycin belong to the macrolide class which have been used as first line drugs for treating camplyobacteriosis (García-Fernández et al., 2018) but azithromycin showed lower MIC values than those of erythromycin against Gram-negative bacteria including *Campylobacter* (Retsema et al., 1987; Wei and Kang, 2018). The WHO has classified fluoroquinolone-resistant *Campylobacter* strains among the pathogens requiring the development of new antibiotics (Zainol et al., 2024). The resistance to STR was higher than 50% reported in Ethiopia (Hagos et al., 2021). Increased resistance to STR has been associated with mutations in the rpsL gene coding for a ribosomal protein (RpsL) or the expression of aminoglycoside-modifying enzyme [ANT(6)-I; Retsema et al., 1987; Wei and Kang, 2018; Dahl et al., 2021; Hormeño et al., 2018]. Resistance of *Campylobacter* isolates from humans and pigs to CIP (25.9–27.0%) was higher than 16% reported in Sub-Saharan Africa (Hlashwayo et al., 2021) and 22.1% reported in Tanzania (Komba et al., 2015). Resistance to CIP has been associated with its misuse in treating diarrhea of unknown etiology and the persistence of its resistance in the community once acquired (Sproston et al., 2018). Resistance to GEN was lower (22.2–25%) among human and pig-derived isolates but higher in chicken isolates (66.9%). Resistance to GEN has been relatively low because it is limited to treating systemic infections (Lynch et al., 2020). Resistance to CHL was relatively low, ranging from 7.4 to 26.9% across the isolates. This suggests that CHL remains a viable option for the treatment of *Campylobacter* infections. Similarly, GEN also exhibited relatively low resistance rates, making it another potential alternative for managing human campylobacteriosis. The use of these antimicrobials could provide effective options, particularly in cases where resistance to first-line treatments such as CIP and ERY is high.

The AMR profiles of *C. jejuni* and *C. coli* isolates were compared. For ERY, *C. jejuni* exhibited higher resistance than *C. coli*, except among isolates from pigs. Conversely, resistance to CIP and STR was higher in *C. coli* than in *C. jejuni*, except for isolates from human stool. Both *C. coli* and *C. jejuni* showed lower resistance to CHL. Resistance to GEN was generally below 50%, except for isolates from chickens. These findings suggest that CHL and GEN could serve as alternative antimicrobials for treating human campylobacteriosis. However, their prudent use is essential to mitigate the risk of increasing resistance.

The present study detected MDR *Campylobacter* isolates with the frequencies of MDR being higher among isolates from chicken. The MDR rates in isolates from pigs and humans were comparable. The frequently occurring MDR combinations were GEN-CIP-ERY and GEN-ERY-STR. Notably, 25% of isolates from chickens, 4.5% from pigs, and 14.8% from humans were resistant to five of the tested antimicrobials. A previous study revealed higher resistance of *Campylobacter* isolates to a combination of fluoroquinolone, tetracycline, and macrolide (Tang et al., 2020). A different study on chicken reported higher resistance to quinolones, tetracycline, and sulfamethoxazole-trimethoprim (Giacomelli et al., 2014). These findings underscore the growing concern over MDR in *Campylobacter* strains across different hosts, highlighting the need for strengthening antimicrobial stewardship programs and the development of alternative treatment options, including phage therapy and the use of natural products.

This study faced a number of limitations. First, the cross-sectional approach could not capture potential changes in prevalence over different seasons or the influence of hygiene and other interventions. Second, the Kirby-Bauer disk diffusion method used is qualitative in nature and could not determine the level of AMR against tested antimicrobials. Last, different protocols were used for *Campylobacter* isolation in human vs. pig and chicken feces. For human feces, enrichment with Preston broth was used while for pig and chicken feces, direct plating onto mCCDA was preferred. This was due to the low intensity of *Campylobacter* in humans compared to animals. Despite the highlighted shortcomings, this study is the first in Rwanda to isolate *Campylobacter* from animal feces by culture and to report antimicrobial profiles. The findings highlight a significant public health threat that should be tackled in a One Health approach.

## Conclusion

The current study highlights higher prevalence of *Campylobacter* in chicken feces when compared to pig and human feces. *Campylobacter jejuni* was the predominant species across the three hosts, but *C. coli* was also identified in a considerable number of fecal samples, mainly from pigs and chicken. An increased resistance of *Campylobacter* isolates to different antimicrobials, including drugs of choice (ERY and CIP) was observed. The study showed that GEN and CHL could be used as alternative treatment options. Further research and development toward non-antibiotic control measures are highly recommended. Genomic studies will provide insights of transmission patterns and specific resistance genes.

## Data Availability

The original contributions presented in the study are included in the article/supplementary material. Further inquiries can be directed to the corresponding authors.

## Footnotes

^1^ www.eucast.org
